# Supplementary material for: Bone-Targeted Delivery of Novokinin as an Alternative Treatment Option for Rheumatoid Arthritis
Source: Pharmaceutics. 2022 Aug 12;14(8):1681. doi: 10.3390/pharmaceutics14081681 (PMC9416659; doi:10.3390/pharmaceutics14081681)

Figure S1. The orthogonal regression graphs with line best fit of RAS components vs ArA metabolites.

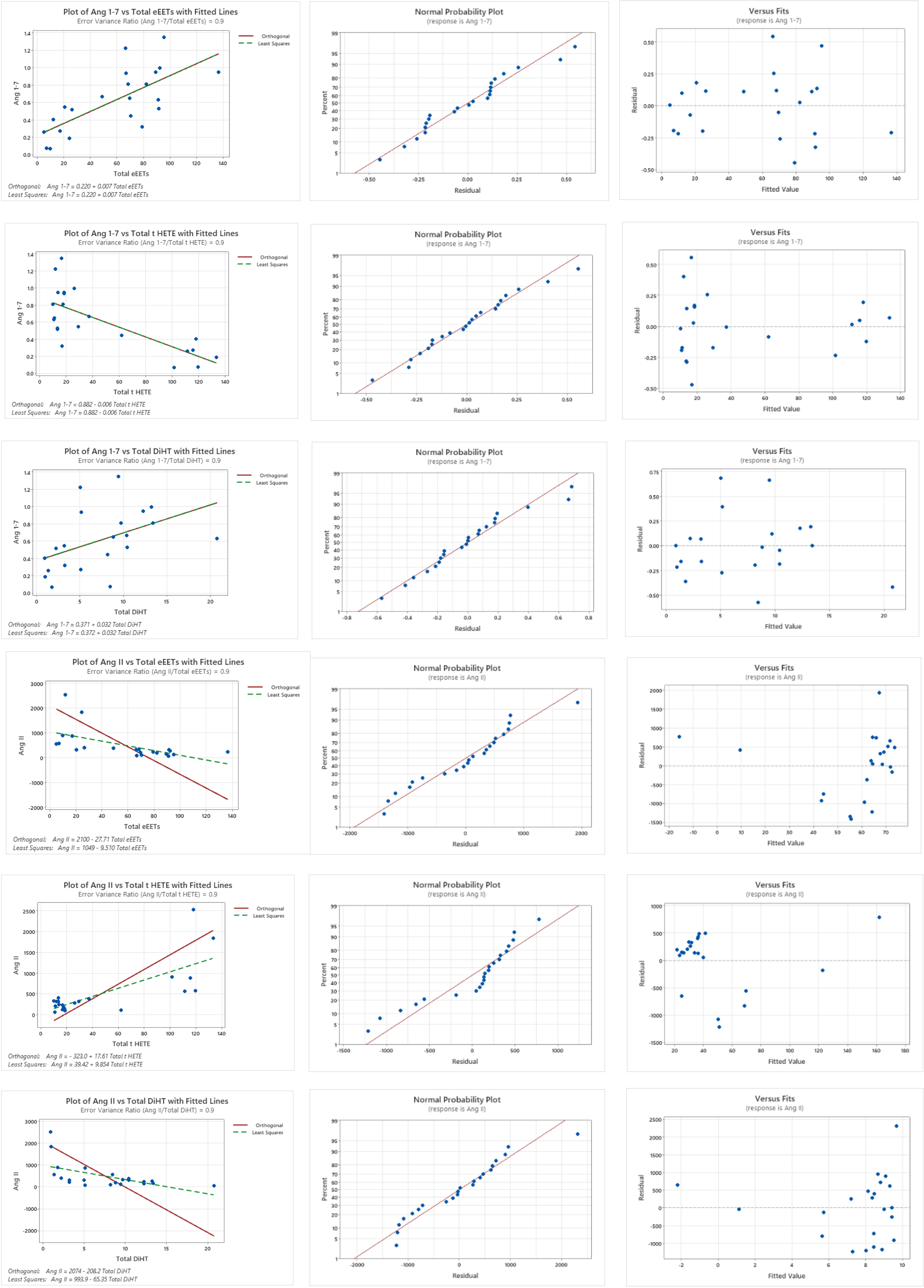

Supplement: Supplementary file 1 [file pharmaceutics-14-01681-s001.zip › Figure S1.pdf]
