# Supplementary material for: Bone-Targeted Delivery of Novokinin as an Alternative Treatment Option for Rheumatoid Arthritis
Source: Pharmaceutics. 2022 Aug 12;14(8):1681. doi: 10.3390/pharmaceutics14081681 (PMC9416659; doi:10.3390/pharmaceutics14081681)
Supplement: Supplementary file 1 [file pharmaceutics-14-01681-s001.zip › Table S1.pdf]

Table S1. Orthogonal regression analysis results.

Predictor	Predictor Coef.	[a]CI of Predictor	[b]SE of Predictor Coef.	Z value	P value	Constant Coef.	[a]CI of Constant	Error in x-axis	Error in y-axis
				Ang 1.7					
11,12-EET	0.012	(0.007; 0.018)	0.003	4.600	0.000	0.246	(0.057; 0.436)	0.068	0.061
14,15-EET	0.019	(0.011; 0.027)	0.004	4.830	0.000	0.252	(0.072; 0.432)	0.064	0.058
Total eEETs	0.007	(0.004; 0.010)	0.001	4.580	0.000	0.220	(0.019; 0.419)	0.068	0.061
19-HETE	-0.110	(-0.160; -0.061)	0.025	-4.338	0.000	0.850	(0.670; 1.002)	0.071	0.064
20-HETE	-0.006	(-0.008; -0.004)	0.001	-4.853	0.000	0.883	(0.734; 1.032)	0.064	0.058
Total t HETE	-0.006	(-0.008; -0.003)	0.001	-4.854	0.000	0.882	(0.733; 1.031)	0.064	0.058
T-EETs/T-HETEs	0.075	(0.036; 0.114)	0.020	3.756	0.000	0.355	(0.176; 0.534)	0.081	0.073
5,6-DiHT	0.247	(0.084; 0.410)	0.083	2.966	0.003	0.361	(0.150; 0.572)	0.091	0.082
8,9-DiHT	1.183	(0.632; 1.734)	0.281	4.208	0.000	-0.006	(-0.321; 0.309)	0.037	0.033
11,12-DiHT	0.070	(0.011; 0.128)	0.030	2.336	0.020	0.392	(0.161; 0.623)	0.109	0.098
Total DiHT	0.032	(0.006; 0.059)	0.014	2.366	0.018	0.371	(0.129; 0.614)	0.109	0.098
				Ang II					
11,12-EET	-52.876	(-89.572; -16.18)	18.723	-2.824	0.005	2076.738	(915.851; 3237.620)	291.826	262.643
14,15-EET	-63.560	(-93.920; -33.200)	15.490	-4.103	0.000	1724.281	(1078.710; 2369.850)	100.672	90.605
Total eEETs	-27.711	(-45.290; -10.130)	8.968	-3.090	0.002	2100.215	(1024.420; 3176.010)	897.738	807.964
19-HETE	426.846	(182.320; 671.370)	124.759	3.421	0.001	-407.324	(-1019.750; 205.100)	3.147	2.833
20-HETE	18.379	(11.181; 25.577)	3.673	5.004	0.000	-319.640	(-711.739; 72.459)	817.982	736.184
Total t HETE	17.615	(10.619; 24.610)	3.569	4.935	0.000	-322.993	(-721.588; 75.603)	914.167	822.750
T-EETs/T-HETEs	-362.518	(-623.971; -101.070)	133.396	-2.718	0.007	1760.554	(766.733; 2754.370)	6.668	6.001
5,6-DiHT	-1372.640	(-2522.930; -222.350)	586.893	-2.339	0.019	1913.940	(643.130; 3184.750)	0.609	0.548
8,9-DiHT	-3301.788	(-5500.940; -1102.640)	1122.04	-2.943	0.003	2232.545	(1019.620; 3445.470)	0.070	0.063
11,12-DiHT	-496.300	(-891.983; -100.620)	201.883	-2.458	0.014	2087.269	(747.642; 3426.900)	4.260	3.834
14,15-DiHT	-569.245	(-1068.590; -69.900)	254.774	-2.234	0.025	2099.332	(617.090; 3581.570)	3.835	3.451
Total DiHT	-208.195	(-349.952; -66.440)	72.326	-2.879	0.004	2074.492	(936.718; 3212.270)	18.206	16.385

[a]95% confidence intervals for each of the regression coefficients. [b]The standard error of a Predictor coefficient.
